# Gallstone ileus following endoscopic retrograde cholangiopancreatography and sphincterotomy: a case report

**DOI:** 10.1186/1752-1947-2-342

**Published:** 2008-11-06

**Authors:** Amit Goyal, Anurag Srivastava

**Affiliations:** 1 Department of Surgery, Wales College of Medicine, Cardiff University, Cardiff, CF14 4XN, UK

## Abstract

**Introduction:**

Gallstone ileus is a mechanical obstruction caused by the impaction of one or more gallstones within the lumen of any part of the gastrointestinal tract. Although the disorder is a rare cause of small bowel obstruction (1% to 2%), it has been reported to cause up to 25% of cases of non-strangulated small bowel obstruction in patients over 65 years of age.

**Case presentation:**

We report a case of a 67-year-old woman who presented with gallstone ileus following endoscopic retrograde cholangiopancreatography and sphincterotomy for choledocholithiasis. She had a history of terminal ileum resection with ileocolic anastomosis for Crohn's disease. A 3 cm gallstone was found to be impacted just proximal to the previous ileocolic anastomosis. A second gallstone was found on digital examination of the proximal small bowel.

**Conclusion:**

A gallstone may enter the gastrointestinal tract following endoscopic retrograde cholangiopancreatography and sphincterotomy and impact proximal to an anastomotic stricture as demonstrated here. The radiographic image of small bowel obstruction plus air in the biliary tree is a classic diagnostic finding. After stone extraction, the entire small bowel and colon should be digitally examined for further stones.

## Introduction

Gallstone ileus, caused by migration of a large gallstone either from the gallbladder or common bile duct into the terminal ileum, is an unusual but well documented cause of intestinal obstruction in the elderly.

## Case presentation

**Figure 1:**
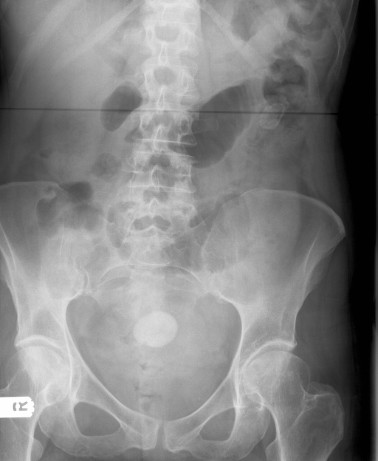
**Abdominal X-ray.** The X-ray shows air in the biliary tree, distended loops of small bowel, and calcified gallstone in the left side of the abdomen.

**Figure 2:**
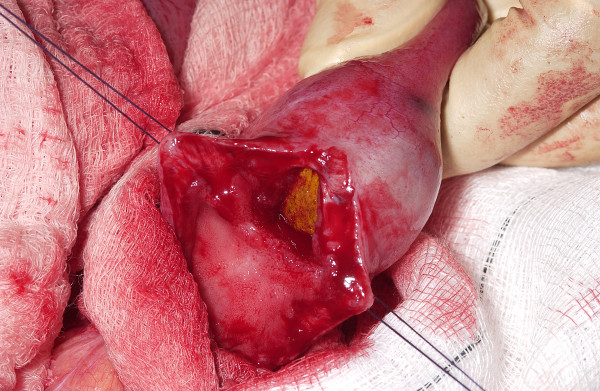
**Enterolithotomy.** The calcified gallstone can be seen in the lumen of the small bowel.

**Figure 3:**
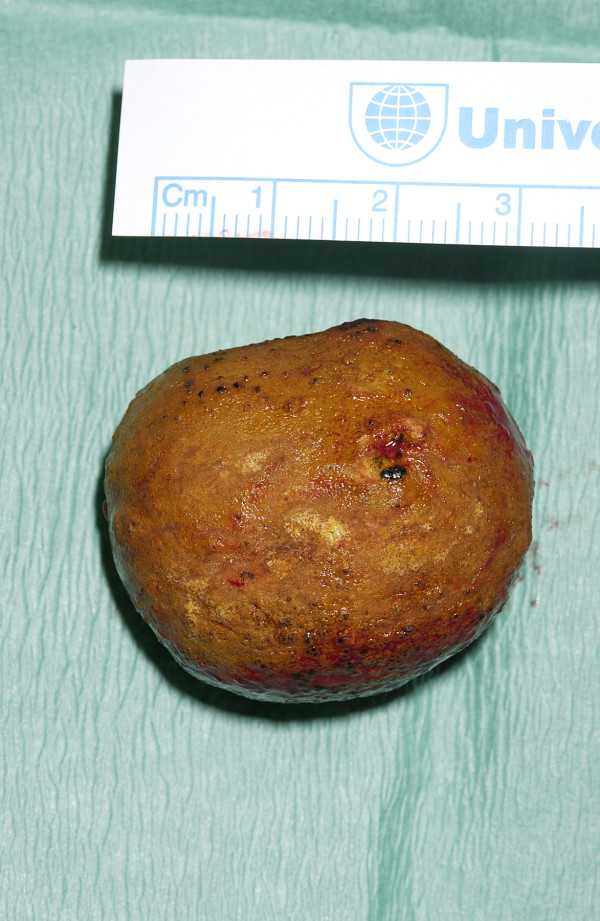
**Gallstone.** The gallstone measured 3 cm in size.

A 67-year-old woman had been unwell for 5 days with intermittent nausea and vomiting, abdominal pain, progressive abdominal distension and obstipation. She had been having similar, though less severe, symptoms for the previous 6 months. She had a history of ERCP for common bile duct stones and terminal ileum resection with ileocolic anastomosis for Crohn's disease. The physical examination revealed a distended abdomen, with no palpable masses and no hernias. Numerous distended loops of small intestine, air in the biliary tree, and a calcified intraluminal mass were identified on the abdominal X-ray (Figure 1). These findings established the diagnosis of gallstone ileus. Laparotomy revealed distended loops of small bowel and a clear transition zone from dilated to collapsed bowel just proximal to the previous ileo-colic anastomosis. A 3 cm stone was palpated at this site. A second gallstone was found on digital examination of the proximal small bowel. The small bowel was healthy with no evidence of active Crohn's disease. The patient underwent enterolithotomy (Figures 2 and 3), and the gallbladder was left in place. A cholecystoduodenal fistula was not found. The patient's postoperative recovery was uneventful.

## Discussion

Gallstone ileus, first described by Dr Erasmus Bartholin in 1654, is a mechanical obstruction caused by the impaction of one or more gallstones within the lumen of any part of the gastrointestinal tract. It is a rare disease and accounts for about 1% to 3% of mechanical ileus of the small bowel, but for 25% of all small bowel obstructions in patients older than 65 years [1]. The gallstone may enter the gastrointestinal tract following endoscopic retrograde cholangiopancreatography (ERCP) and sphincterotomy (as seen in this patient) or more commonly through a fistula; normally between a gangrenous gallbladder and the duodenum (cholecystoduodenal), but occasionally between the gallbladder and other parts of the gastrointestinal tract or between the bile duct and duodenum (choledochoduodenal). The most common site of impaction of the stone is the distal ileum, followed by the jejunum and stomach [2]. A gallstone may be impacted proximal to a stricture (anastomotic or inflammatory) as demonstrated here. Gallstones which obstruct the intestinal lumen are usually greater than 2.5 cm in diameter. The pre-operative diagnosis of gallstone ileus is often inaccurate, sometimes because of the intermittent nature of the small bowel obstruction.

Plain abdominal X-rays may reveal signs of small bowel obstruction, calcified gallstone in the intestinal lumen (less than 15% of gallstones are radiopaque) or air in the biliary system (pneumobilia). Computed tomography (CT) scans may also provide useful additional information if the diagnosis is in doubt. CT is useful for estimating the size and number of impacted gallstones and the transition point between dilated and collapsed bowel [3]. Magnetic resonance cholangiopancreatography (MRCP) can demonstrate air in the biliary tree but has limited value in diagnosing the site and cause of small bowel obstruction.

Enterolithotomy is usually curative. The approach to the biliary enteric fistula is arguable and somewhat contingent upon the patient's clinical status [4]. A one-stage procedure includes an enterolithotomy, cholecystectomy and fistula closure. The two-stage strategy prefers an enterolithotomy as an emergency operation, followed by cholecystectomy in an inflammatory-free interval 4 to 6 weeks later.

## Conclusion

This case report demonstrates that gallstones may enter the gastrointestinal tract following ERCP and sphincterotomy and impact proximal to an anastomotic stricture. The radiographic image of small bowel obstruction plus air in the biliary tree is a classic diagnostic finding. After stone extraction, the entire small bowel and colon should be digitally examined for further stones.

## Abbreviations

CT: computed tomography; ERCP: endoscopic retrograde cholangiopancreatography; MRCP: magnetic resonance cholangiopancreatography.

## Consent

Written informed consent was obtained from the patient for publication of this case report and any accompanying images. A copy of the written consent is available for review by the Editor-in-Chief of this journal.

## Competing interests

The authors declare that they have no competing interests.

## Authors' contributions

AG and AS contributed to the writing and editing of the manuscript. Both authors read and approved the final manuscript.
